# Sustainable recovery of copper from wastewater via zinc cementation enhanced by natural chalcone-based accelerators

**DOI:** 10.1038/s41598-025-16475-7

**Published:** 2025-08-25

**Authors:** Ashraf Morsy, Hanaa H. Abdel Rahman, Hesham M. Kamel, Hassan A. Ewais, Abd El Monaem M. Ahmed, Ahmed Morsy, Eman A. Fadl, Elsayed G. Blall

**Affiliations:** 1 Faculty of Engineering, Petrochemical Department, Pharos University in Alexandria, Canal El Mahmoudia Street, Beside Green Plaza Complex, , Alexandria, 21648 Egypt; 2 Chemistry Department, Faculty of Science, Alexandria University, Alexandria, Egypt; 3 Department of Medical Laboratory Technology, Faculty of Allied Medical Sciences, Pharos University, Alexandria, Egypt; 4 Chemistry Department, Faculty of Science, King Abdulaziz University, P.O. Box 80203, 21589 Jeddah, Saudi Arabia; 5 Chemistry Department, Faculty of Science, Beni-Suef University, P.O. Box 62514, Beni-Suef, Egypt; 6 Faculty of Dentistry and Oral Surgery, Pharos University in Alexandria, Alexandria, Egypt; 7 Institute of Graduate Studies and Research, Alexandria University, Alexandria, Egypt

**Keywords:** Copper recovery, Zinc cementation, Chalcone derivatives, Green accelerators, Wastewater treatment, Biocatalysis, Metals, Chemistry, Engineering, Materials science, Physics

## Abstract

This study presents an eco-friendly strategy to enhance copper ion recovery from industrial wastewater through zinc cementation, utilizing natural chalcone-derived pyrazolo-quinoxaline compounds as organic accelerators. These bio-based additives notably improve recovery efficiency by promoting dendritic copper deposition, which enhances surface reactivity and mass transfer rates. For the first time, this research combines quantum chemical analysis with a dimensionless model (Sh = 0.81Sc^0.33Re^0.47) to explain the mechanism of chalcone-assisted cementation, proposing a sustainable and mechanistically grounded approach for metal recovery.

## Introduction

Heavy metals are among the most hazardous pollutants in industrial wastewater, with copper being a particularly common contaminant due to activities such as metal finishing, electroplating, and mining^1^. As a non-biodegradable element that accumulates in aquatic organisms, copper poses serious risks to both ecosystems and human health. This highlights the urgent need for efficient and sustainable recovery methods^2^. Addressing heavy metal contamination in waste streams and low-grade ores remains a major challenge in modern hydrometallurgy, where innovative approaches are required to recover valuable metals while minimizing environmental^3^. Traditional recovery methods such as solvent extraction, ion exchange, precipitation, and electrochemical deposition are widely used, but they often suffer from high operational costs, energy consumption, or the use of hazardous chemicals^4^. Moreover, many of these techniques require complex separation stages, limiting their practicality in treating multi-metal or dilute solutions^5^. Cementation, a spontaneous redox reaction where a more electropositive metal (e.g., zinc) reduces metal ions (e.g., Cu^2+^) from solution, has emerged as a cost-effective and simple alternative. The process does not require an external power source and operates efficiently under ambient conditions^6^. Its industrial appeal lies in its scalability, low energy input, and rapid kinetics in favorable systems^7^. However, limitations such as surface passivation, poor selectivity, and reaction rate decline due to diffusion barriers remain concerns that hinder broader implementation^8^. To address these challenges, researchers have explored the use of organic additives to accelerate the cementation process, modulate deposit morphology, and improve reaction kinetics^9^. Many reported additives are synthetic and pose toxicity or disposal concerns, contradicting the increasing demand for sustainable and environmentally benign technologies. In this context, naturally derived organic molecules offer a promising alternative due to their biodegradability, low toxicity, and tunable chemical properties^10^.

Chalcones, a class of α, β-unsaturated ketones naturally occurring as biosynthetic precursors of flavonoids and isoflavonoids, have attracted attention as green additives in metal recovery processes^11–14^. These molecules consist of two aromatic rings linked by a conjugated enone bridge, providing a versatile scaffold for coordination with metal ions^15–17^. They are known for their rich electron-donating capacity, ability to form stable complexes, and wide range of biological and catalytic activities^18^. Furthermore, their structure allows for functional modifications such as halogen or hydroxyl substitutions that can enhance redox reactivity and metal selectivity^19^. Recent studies have explored functionalized chalcones and their heterocyclic derivatives, such as pyrazolo-quinoxalines, in catalytic and coordination chemistry^20–22^. These compounds exhibit strong chelating behavior, improved thermal stability, and favorable electronic properties, making them attractive candidates for promoting redox reactions like cementation^23^. However, their application in metal recovery particularly in heterogeneous systems like zinc-copper cementation remains underexplored^24–26^. In this study, we investigate a series of pyrazolo-quinoxaline derivatives synthesized from chalcones as eco-friendly organic accelerators in the cementation of Cu^2+^ onto zinc in a stirred aqueous system. We analyze the influence of additive concentration, temperature, and stirring speed on the deposition rate and metal morphology. Furthermore, thermodynamic activation parameters are calculated to gain insight into the underlying reaction mechanisms. This work contributes to the development of sustainable strategies for metal recovery by combining green chemistry principles with kinetic and morphological analysis.

## Materials and methods

### Materials

Copper sulfate pentahydrate (CuSO_4_·5H_2_O, analytical grade) was obtained from Adwic Chemicals and used to prepare Cu^2+^ stock solutions. Zinc sheets (99.9% purity) were purchased from J.T. Baker Inc., with dimensions of 7.5 cm × 3.33 cm × 0.15 cm. Sulfuric acid was obtained from Fisher Scientific and a series of bio-derived chalcone-based pyrazolo-quinoxaline derivatives were used as organic additives. These include: (a) 1-phenyl-3-(1-phenylpyrazolo[3,4-b]quinoxalin-3-yl)phenyl-2-propenone, (b) 1-phenyl-3-(1-phenylpyrazolo[3,4-b]quinoxalin-3-yl)-p-tolyl-2-propenone, (c) 1-(4-chlorophenyl)-3-(1-phenylpyrazolo[3,4-b]quinoxalin-3-yl)-2-propenone, (d) 1-(4-bromophenyl)-3-(1-phenylpyrazolo[3,4-b]quinoxalin-3-yl)-2-propenone, (e) 1-(biphenyl)-3-(1-phenylpyrazolo[3,4-b]quinoxalin-3-yl)-2-propenone.

### Methods

#### Synthesis and characterization of additives

The synthesis and characterization of the chalcone-based pyrazolo-quinoxaline derivatives (compounds a-e) were conducted following previously reported procedures by Wei et al.^13^ and Karaca and Kazancı^14^. Briefly, chalcone intermediates were obtained via Claisen-Schmidt condensation of selected acetophenones with aromatic aldehydes under basic conditions, followed by cyclization with phenylhydrazine and *o*-phenylenediamine to form the final pyrazolo-quinoxaline scaffold. The structures of the synthesized compounds were confirmed using nuclear magnetic resonance (NMR) and mass spectrometry (MS), as described by Amarasekara^18^.

#### Preparation of copper solutions

A stock solution of 1000 ppm CuSO_4_.5H_2_O was freshly prepared using distilled water prior to each experiment to ensure stability. The pH of the copper solution was adjusted to 5.0 ± 0.1 using dilute H_2_SO_4_. This pH was selected to maintain copper predominantly in the Cu^2+^ ionic form, minimizing hydrolysis and preventing precipitation of Cu(OH)_2_, consistent with known aqueous copper chemistry under mildly acidic conditions. Buffer solutions were deliberately avoided to eliminate potential interactions between buffer species and metal ions that might interfere with copper speciation or the cementation process. All solutions were prepared with deionized water, and while the ionic strength was not independently controlled, it was kept consistent across all experiments.

#### Effect of additive concentration

The organic additives (a-e) were tested at six different concentrations: 3.0 × 10⁻⁷, 3.0 × 10⁻⁶, 6.0 × 10⁻⁶, 1.2 × 10⁻⁵, 2.4 × 10⁻⁵, and 3.0 × 10⁻⁵ mol/L, selected to cover a broad range relevant for evaluating surface inhibition effects.

#### Effect of temperature

Unless otherwise specified, all experiments were conducted at 25 °C, which is standard temperature favoring stable reaction kinetics and reproducible results in cementation studies^13,14^.

#### Effect of agitation speed

Zinc sheets (99.9% purity) were mechanically polished using P150-grade emery paper, degreased with trichloroethylene, rinsed with ethanol, and washed with distilled water to ensure a clean and reactive surface. For each experiment, 500 mL of the copper solution (with or without additives) was transferred to a glass beaker, and a zinc sheet was vertically immersed. Agitation was applied using a magnetic stirrer (Medline MS 300 HS) at controlled speeds of 100, 200, 500, 750, and 1000 rpm. Standard conditions unless noted were 750 rpm and a reaction time of 60 min.

#### Sampling and analysis

**Fig. 1:**
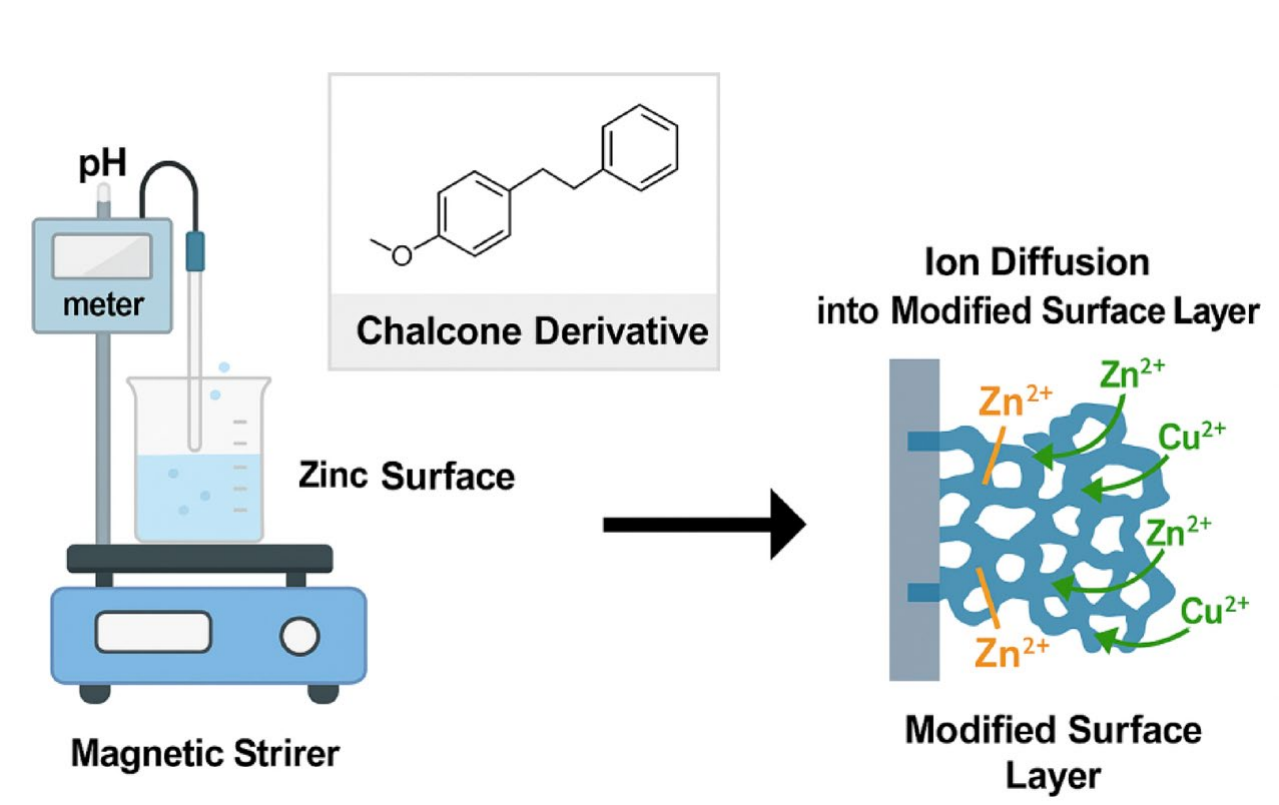
Schematic of the cementation reaction mechanism between Cu^2+^ ions and zinc surface showing electron transfer and copper deposition.

Cementation progress was monitored in the presence and absence of additives. At 5, 10, 15, 20, 25, and 30 min, 0.5 mL aliquots were withdrawn and immediately diluted with 5 mL of distilled water for analysis. All sampling was performed under constant pH and ionic strength to maintain stable Cu^2+^ speciation. While pH and ionic strength can influence copper chemistry and cementation behavior, these controlled conditions minimize their impact, allowing redox kinetics and diffusion between Zn⁰ and Cu^2+^ to be the dominant factors as shown in Fig. 1.

### Characterization

The concentration of Cu^2+^ ions in solution was determined using a HITACHI 180 − 50 atomic absorption spectrophotometer (AAS). Measurements were carried out at a wavelength of 324.8 nm using an air-acetylene flame and a slit width of 0.5 nm. Standard calibration solutions were prepared in the concentration range of 0.1 to 5.0 mg/L, and a linear calibration curve was obtained with a correlation coefficient (R²) greater than 0.999. The limit of detection (LOD) and limit of quantification (LOQ) were found to be 0.02 mg/L and 0.06 mg/L, respectively. All measurements were conducted in triplicate to ensure precision and reproducibility. Viscosity measurements were conducted using a Koehler K23400 viscometer bath, while the diffusion coefficient was obtained via the capillary method. Morphological analysis of copper deposits was performed using a Cambridge Stereo scan 360 scanning electron microscope (SEM), with ten representative samples examined. Elemental composition and confirmation of copper deposition were further supported by energy-dispersive X-ray spectroscopy (EDX). Quantum chemical calculations were performed using the MOPAC 2009 Public Domain Version. The molecular geometries of the pyrazolo-quinoxaline derivatives were fully optimized using semi-empirical methods, specifically AM1 and PM3. These methods were selected for their computational efficiency and reasonable accuracy in predicting electronic properties of organic heterocyclic compounds, as reported in prior studies^27^. After geometry optimization, key quantum descriptors were calculated, including the energies of the highest occupied molecular orbital (E_HOMO_), the lowest unoccupied molecular orbital (E_LUMO_), the energy gap (ΔE = E_LUMO_ − E_HOMO_), and the dipole moment (µ). These parameters provide insights into the electronic structure, chemical reactivity, and electron transfer capability of the compounds. According to Koopmans’ theorem^28,29^ the ionization potential (I) and electron affinity (A) can be approximated as following Eqs. (1, 2):

1 $$I={\text{ }} - {E_\text{HOMO}}$$

2 $$A={\text{ }} - {E_\text{LUMO}}$$

## Results and discussion

### Effect of initial copper ion concentration

The initial copper ion concentration plays a key role in determining the cementation rate. Experiments were conducted using Cu^2+^ concentrations of 50, 75, 100, 250, and 500 ppm. The kinetics was evaluated using the following Eq. (3)^29^:

3 $$\:Log\left(\frac{C_{0}}{C}\right)=\left(\frac{a\times\:\:k}{2.303V}\right)\times\: t$$

Where *a* is the zinc surface area (cm²), *V* is the solution volume (cm^3^), *k* is the mass transfer coefficient (cm/s), *C₀* is the initial copper concentration, and *C* is the concentration at time *t*.

**Fig. 2:**
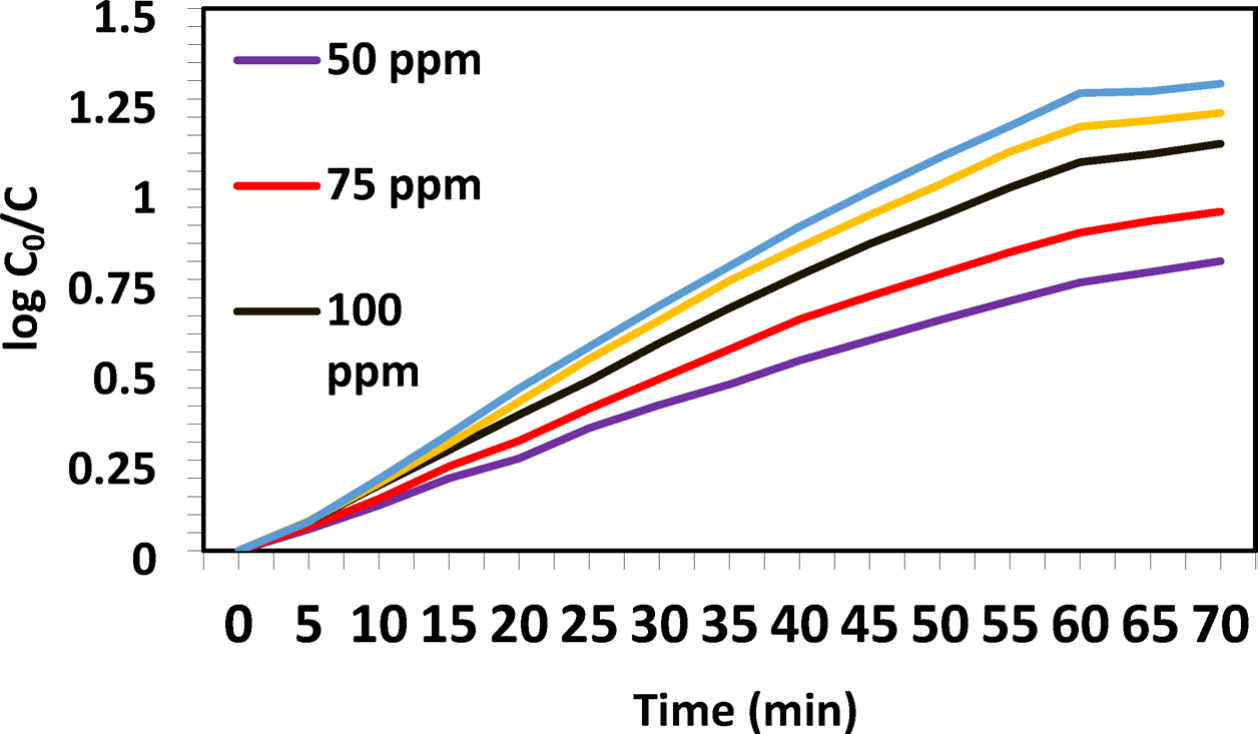
Kinetic curves of the copper cementation reaction by zinc at 25 °C and 750 rpm for different Cu^2+^ concentrations.

Figure 2 show that the experimental data fit Eq. (3) well. The slope of the log (*C₀*/*C*) vs. time plot was used to calculate *k*, with results summarized in Table 1. The results showed that the rate of copper removal increased with higher concentrations. This is due to the increased driving force for mass transfer from the solution to the zinc surface. However, at concentrations above 250 ppm, the increase in rate was less significant. This may be attributed to the formation of dense copper layers that hinder further diffusion. As Cu^2+^ concentration increases, the mass transfer coefficient also increases due to enhanced ion diffusion and electrostatic interactions at the zinc surface. Consequently, higher concentrations accelerate the cementation rate, leading to larger copper deposits. These findings confirm that the process is governed by diffusion the primary cementation reaction obtained from Eq. (4):

4 $${\text{Zn}}\,+\,{\text{C}}{{\text{u}}^{{\text{2+}}}} \to {\text{Cu}}\,+\,{\text{Z}}{{\text{n}}^{2+}}$$

In industrial applications, copper is recovered by passing leach solutions over scrap zinc, where Cu^2+^ is reduced to Cu. Although this method is simple, the final copper product may require purification. The overall cementation process involves coupled mass and charge transfer steps:

5 $${\text{C}}{{\text{u}}^{{\text{2+}}}}_{{({\text{bulk}})}} \to {\text{ C}}{{\text{u}}^{{\text{2+}}}}_{{({\text{interface}})}}~~\left( {{\text{mass transfer}}} \right)$$

6 $${\text{Zn }} \leftrightarrow {\text{ Z}}{{\text{n}}^{{\text{2+}}}}_{{({\text{interface}})}}+{\text{ }}2{\text{e}}^{-}\;\;~\left( {{\text{charge transfer}}} \right)$$

7 $${\text{Z}}{{\text{n}}^{{\text{2+}}}}_{{({\text{interface}})}} \to {\text{ Z}}{{\text{n}}^{{\text{2+}}}}_{{\left( {{\text{bulk}}} \right)~}}\left( {{\text{mass transfer}}} \right)$$

8 $${\text{C}}{{\text{u}}^{{\text{2+}}}}_{{({\text{interface}})}}+{\text{ 2}}{{\text{e}}^-}{\text{ }} \to {\text{ Cu}}~~\left( {{\text{charge transfer}}} \right)$$

The rate-determining step can be either mass or charge transfer. In this study, the reaction was found to be diffusion-controlled, as indicated by the concentration-dependent increase in mass transfer coefficients.

**Table 1:** Mass transfer coefficients (cm/s) at different Cu^2+^ concentrations (ppm) measured at 25 °C and 750 rpm.

Conc. ppm	50	75	100	250	500
k x 10^3^ (cm/s)	4.91	5.87	7.10	7.85	8.36

### Effect of rotation speed

Stirring enhances the mass transfer of Cu^2+^ ions to the zinc surface at a constant temperature of 25 °C. As stirring speed increased from 300 to 900 rpm, the cementation rate also improved. Higher agitation reduces the thickness of the diffusion layer and allows faster ion transport.

**Fig. 3:**
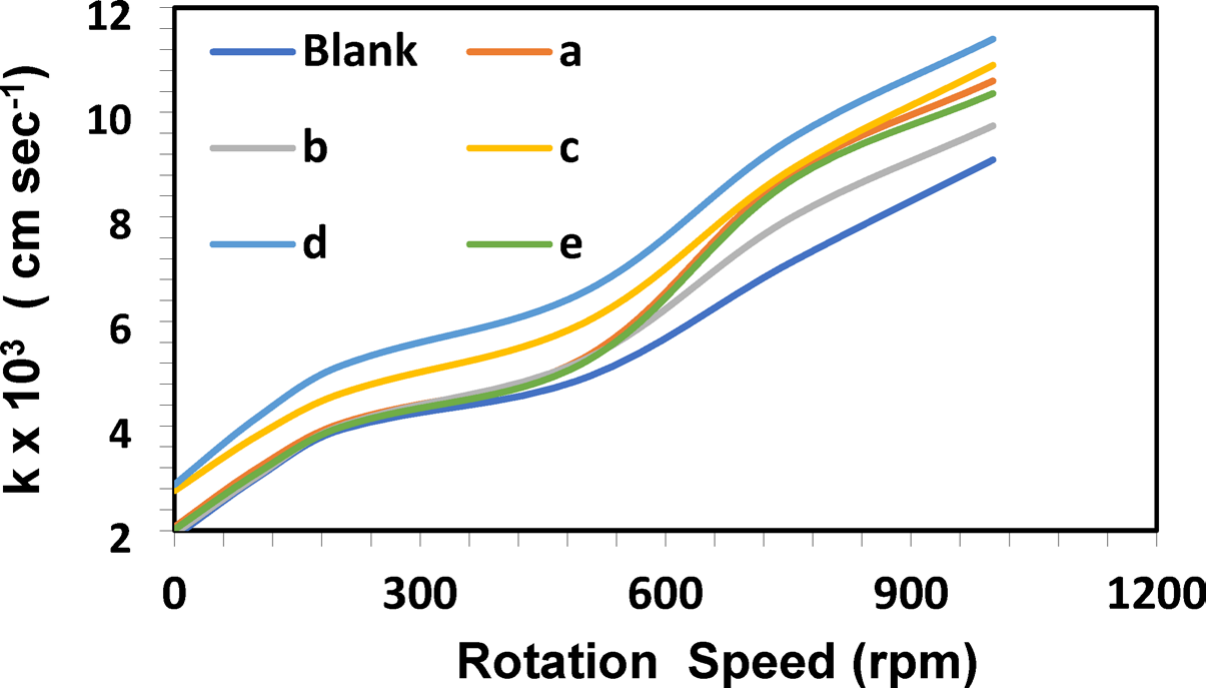
Effect of rotation speed (rpm) on the mass transfer coefficient with and without Chalcones (3 × 10^− 7^ mol L^−1^) for 100 ppm Cu^2+^ at 25 °C.

The relationship between stirring speed and mass transfer coefficient followed a typical hydrodynamic behavior, confirming that the reaction is controlled by diffusion. The relationship between log (*C₀/C*) and time at various stirring rates is presented in Table 2; Fig. 3. The results show that the cementation rate increases with increasing rotation speed. This enhancement is attributed to the reduction in both the diffusion layer thickness (δ) and the hydrodynamic boundary layer on the zinc surface. A thinner diffusion layer facilitates faster ion transport, thereby increasing the mass transfer coefficient. Since Cu^2+^ ions must diffuse through this layer to reach the zinc surface, a reduced δ leads to a higher deposition rate of copper^30^.

**Table 2:** Effect of rotation speed (rpm) on the mass transfer coefficient (k × 10^3^ cm/s) for Chalcones at 25 °C and 100 Ppm cu^2+^.

Speed (rpm)	k x 10^3^ (cm/s)					
	Blank	a	b	c	d	e
0	1.88	2.07	1.94	2.76	2.88	2.02
100	3.00	3.18	3.03	3.80	4.15	3.09
200	3.91	4.03	3.98	4.60	5.13	3.96
500	4.91	5.30	5.26	5.97	6.56	5.21
750	7.10	8.79	7.95	8.87	9.46	8.66
1000	9.09	10.60	9.74	10.90	11.40	10.36

### The impact of temperature

**Fig. 4:**
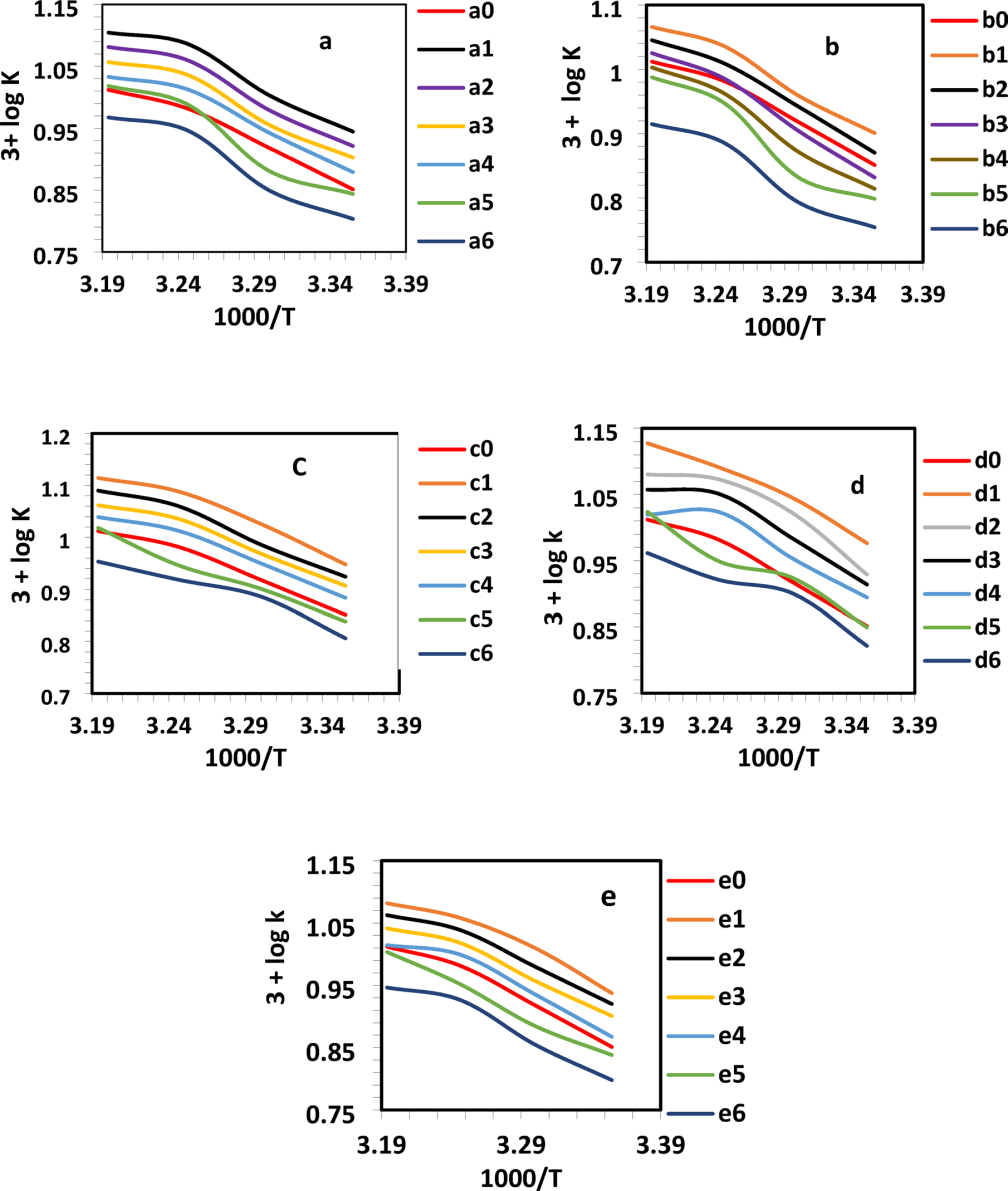
Relationship between log k and 1000/T for 100 ppm Cu^2+^ solution at 750 rpm with varying concentrations of chalcones (a-e).

The cementation process was evaluated at temperatures of 25, 30, 35, and 40 °C, maintaining a constant stirring speed of 750 rpm and an initial Cu^2+^ concentration of 100 ppm. Temperature plays a critical role in influencing both the reaction kinetics and the underlying mechanism. As shown in Table 3; Fig. 4, the mass transfer coefficient increases with rising temperature, indicating enhanced cementation rates. This enhancement is primarily attributed to the temperature-induced changes in physical properties of the solution specifically, a decrease in viscosity and an increase in the diffusion coefficient (D). These changes facilitate faster transport of Cu^2+^ ions to the zinc surface, thereby accelerating the cementation process^31^.

**Table 3:** Mass transfer coefficient (k × 10^3^ cm s^−1^) values for Cu^2+^ at varying temperatures and in the presence of different chalcone concentrations (a-e).

t °C	a_0_	a_1_	a_2_	a_3_	a_4_	a_5_	a_6_
	Blank	3.0 × 10^− 7^	3.0 × 10^− 6^	6.0 × 10^− 6^	1.2 × 10^− 5^	2.4 × 10^− 5^	3.0 × 10^− 5^
25 °C	7.10	8.80	8.34	7.99	7.57	6.98	6.36
30 °C	8.29	10.07	9.53	9.02	8.76	7.60	7.07
35 °C	9.59	12.20	11.48	10.87	10.30	9.74	8.84
40 °C	10.29	12.72	12.06	11.40	10.79	10.43	9.28

t °C	b_0_	b_1_	b_2_	b_3_	b_4_	b_5_	b_6_
	Blank	3.0 × 10^− 7^	3.0 × 10^− 6^	6.0 × 10^− 6^	1.2 × 10^− 5^	2.4 × 10^− 5^	3.0 × 10^− 5^
25 °C	7.10	7.96	7.42	6.79	6.52	6.29	5.68
30 °C	8.29	9.10	8.75	8.02	7.44	6.79	6.21
35 °C	9.59	10.87	10.22	9.68	9.18	8.87	7.69
40 °C	10.29	11.64	11.10	10.60	10.07	9.72	8.22

t °C	c_0_	c_1_	c_2_	c_3_	c_4_	c_5_	c_6_
	Blank	3.0 × 10^− 7^	3.0 × 10^− 6^	6.0 × 10^− 6^	1.2 × 10^− 5^	2.4 × 10^− 5^	3.0 × 10^− 5^
25 °C	7.1	8.87	8.40	8.07	7.65	6.89	6.40
30 °C	8.29	10.61	9.68	9.30	8.91	7.96	7.69
35 °C	9.60	12.26	11.49	10.86	10.30	8.84	8.29
40 °C	10.29	13.01	12.30	11.53	10.94	10.43	8.98

t °C	d_0_	d_1_	d_2_	d_3_	d_4_	d_5_	d_6_
	Blank	3.0 × 10^− 7^	3.0 × 10^− 6^	6.0 × 10^− 6^	1.2 × 10^− 5^	2.4 × 10^− 5^	3.0 × 10^− 5^
25 °C	7.1	9.47	8.49	8.20	7.84	7.07	6.63
30 °C	8.29	11.10	10.53	9.64	8.99	8.40	7.96
35 °C	9.60	12.34	11.83	11.25	10.56	8.90	8.34
40 °C	10.29	13.41	12.03	11.41	10.46	10.56	9.15

t °C	e_0_	e_1_	e_2_	e_3_	e_4_	e_5_	e_6_
	Blank	3.0 × 10^− 7^	3.0 × 10^− 6^	6.0 × 10^− 6^	1.2 × 10^− 5^	2.4 × 10^− 5^	3.0 × 10^− 5^
25 °C	7.1	8.66	8.32	7.96	7.37	6.89	6.28
30 °C	8.29	10.23	9.55	9.06	8.62	7.67	7.16
35 °C	9.60	11.44	10.94	10.43	9.99	8.96	8.45
40 °C	10.29	12.07	11.55	11.00	10.34	10.08	8.84

**Table 4:** Diffusion coefficient and viscosity of a 100 ppm Cu^2+^ solution at 25°C with varying chalcone concentrations.

Chalcones	a		b		c		d		e	
Conc. Mol L^−1^	η	D	η	D	η	D	η	D	η	D
3.0 × 10^− 7^	1.0010	7.26	1.014	7.26	1.007	7.28	1.002	7.32	1.012	7.24
3.0 × 10^− 6^	0.9995	6.66	1.014	6.66	1.07	6.85	1.05	6.98	1.11	6.60
6.0 × 10^− 6^	0.9998	6.27	1.014	6.27	1.15	6.37	1.12	6.55	1.18	6.21
1.2 × 10^− 5^	0.9978	5.68	1.014	5.68	1.27	5.77	1.25	5.86	1.3	5.64
2.4 × 10^− 5^	0.9955	5.24	1.014	5.24	1.38	5.31	1.34	5.47	1.41	5.20
3.0 × 10^− 5^	0.9936	4.58	1.014	4.58	1.55	4.73	1.52	4.82	1.61	4.55

η: Viscosity (g cm^− 1^ s^− 1^), D: Diffusion × 10^6^ (cm^2^ s^− 1^).

Table 5 presents the apparent activation energy (*Ea*) calculated using the Arrhenius equation. These values align with a first-order reaction controlled by diffusion in solution^32–34^. The following equations were applied to determine activation energy (*Ea*, kJ mol^−1^), enthalpy of activation (ΔH*, kJ mol^−1^), entropy of activation (ΔS*, *J* mol^−1^ K^−1^), and Gibbs free energy of activation (ΔG*, kJ mol^−1^):

9 $$\Delta {H^*}={E_a} - {\text{ R}}T$$

10 $$\:\varDelta\:\text{S}\text{*}/\text{R}\hspace{0.17em}=\hspace{0.17em}\text{l}\text{n}\left(A\right)\:-\:\text{l}\text{n}\left(\frac{\alpha\:Te}{h}\right)$$

11 $$\Delta {G^*}={\text{ }}\Delta {H^*} - {\text{ T}}\Delta {S^*}$$

where *A* is the pre-exponential factor related to concentration, steric effects, and surface properties; α is the Boltzmann constant; e ≈ 2.7183; h is Planck’s constant; and R is the universal gas constant. Table 5 shows the activation parameters of the cementation process. Chalcone concentration slightly affects *ΔG*′; *ΔH*′ and *ΔS*′ compensate, causing minor changes in *ΔG*′^35^. The highly negative ΔS* values indicate a more ordered system and nonrandom chalcone distribution on the electrode surface. Despite influencing zinc dissolution, chalcones do not significantly alter these thermodynamic parameters^36^.

**Table 5:** Thermodynamic parameters and activation energy for a 100 ppm Cu^2+^ solution containing Chalcones at 750 rpm.

	Conc. molL^− 1^	E_a_ (kj mol^− 1^)	∆S*(Jmol^− 1^ K^− 1^)	∆H*(kJmol^− 1^)	∆G *(kJmol^− 1^)
	Blank	19.59 ± 2.00	− 228.52 ± 6.57	17.11 ± 2.01	85.24 ± 3.96
a	3.0 × 10^− 7^	20.19 ± 3.08	− 224.78 ± 10.00	17.70 ± 3.08	84.72 ± 6.09
	3.0 × 10^− 6^	20.11± 2.79	− 225.49 ± 9.14	17.63 ± 2.80	84.86 ± 5.51
	6.0 × 10^− 6^	19.48 ± 2.76	− 227.97 ± 9.03	17.00 ± 2.76	84.96 ± 5.45
	1.2 × 10^− 5^	19.07 ± 2.66	− 229.72 ± 8.74	16.59 ± 2.67	85.07 ± 5.28
	2.4 × 10^− 5^	22.56± 3.77	− 218.94 ± 12.33	20.09 ± 3.76	85.36 ± 7.44
	3.0 × 10^− 5^	21.09 ± 3.42	− 224.55 ± 11.21	18.61± 3.42	85.56 ± 6.77
b	3.0 × 10^− 7^	20.49 ± 2.19	− 224.62 ± 7.15	18.01 ± 2.18	84.98 ± 4.32
	3.0 × 10^− 6^	21.21 ± 1.93	− 222.72 ± 6.24	18.73 ± 1.93	85.14 ± 3.81
	6.0 × 10^− 6^	23.70 ± 2.08	− 215.14 ± 6.80	21.23 ± 2.08	85.37 ± 4.10
	1.2 × 10^− 5^	23.53 ± 2.27	− 216.18 ± 7.43	21.05 ± 2.27	85.50 ± 4.48
	2.4 × 10^− 5^	24.41 ± 4.10	− 213.69 ± 13.33	21.93 ± 4.10	85.64 ± 8.05
	3.0 × 10^− 5^	20.54 ± 3.61	− 227.40 ± 9.83	18.06 ± 3.00	85.86 ± 5.93
c	3.0 × 10^− 7^	20.15 ± 2.79	− 224.73 ± 9.12	17.67 ± 2.78	84.67 ± 5.50
	3.0 × 10^− 6^	20.46± 2.16	− 224.25 ± 7.08	17.98 ± 2.16	84.84 ± 4.28
	6.0 × 10^− 6^	19.07 ± 2.19	− 229.23 ± 7.11	16.59 ± 2.17	84.93 ± 4.29
	1.2 × 10^− 5^	18.95 ± 2.23	− 230.02 ± 7.31	16.48 ± 2.23	85.06 ± 4.42
	2.4 × 10^− 5^	20.93± 1.32	− 224.41 ± 4.31	18.45 ± 1.32	85.36 ± 2.60
	3.0 × 10^− 5^	16.99± 2.67	− 237.98 ± 8.75	14.51 ± 2.67	85.47 ± 5.28
d	3.0 × 10^− 7^	17.88 ± 1.7	− 231.81 ± 5.58	15.41 ± 1.70	84.52 ± 3.36
	3.0 × 10^− 6^	18.14± 4.67	− 231.64 ± 15.3	15.66 ± 4.68	84.72 ± 9.26
	6.0 × 10^− 6^	17.86± 3.73	− 233.02 ± 15.43	15.38 ± 3.74	84.86 ± 7.39
	1.2 × 10^− 5^	16.00 ± 4.04	− 239.65 ± 13.22	13.52 ± 4.04	84.97 ± 7.98
	2.4 × 10^− 5^	19.58 ± 2.50	− 228.63 ± 8.19	17.10 ± 2.50	85.27 ± 4.95
	3.0 × 10^− 5^	15.78± 2.86	− 241.77 ± 9.37	13.30 ± 2.86	85.38 ± 5.65
e	3.0 × 10^− 7^	17.25 ± 2.59	− 234.61 ± 8.49	14.77 ± 2.59	84.72 ± 5.13
	3.0 × 10^− 6^	17.43± 2.08	− 234.45 ± 6.76	14.95 ± 2.07	84.85 ± 4.08
	6.0 × 10^− 6^	17.28± 2.01	− 235.32 ± 6.58	14.81 ± 2.01	84.97 ± 3.97
	1.2 × 10^− 5^	18.12 ± 3.65	− 233.07 ± 9.94	15.64 ± 3.02	85.13 ± 6.00
	2.4 × 10^− 5^	20.13± 1.05	− 227.16 ± 3.45	17.65 ± 1.06	85.38 ± 2.09
	3.0 × 10^− 5^	18.54± 2.56	− 233.09 ± 8.40	16.06 ± 2.56	85.56 ± 5.07

*Ea*: Activation energy (kJmol^− 1^), ΔH^*^: Enthalpy of activation (kJmol^− 1^), ΔS^*^: Entropy of activation (Jmol^− 1^ K^− 1^), ΔG^*^: Free energy of activation (kJmol^− 1^).

### Impact of chalcone derivatives on the cementation process

The addition of chalcone derivatives significantly improved the cementation rate. Among the tested compounds (a-e), derivative D exhibited the highest acceleration, with a 47.6% increase in copper removal compared to the blank. This enhancement is attributed to the ability of chalcones to coordinate with Cu^2+^ ions, facilitating their transport and reduction on the zinc surface. The variation in performance between derivatives can be linked to differences in their molecular structures and charge distribution, as discussed below. Table 6 shows that the reaction rate initially increases with chalcone derivative concentration, reaching a maximum before decreasing as concentration continues to rise (compounds a-e). At low chalcone concentrations, solution viscosity remains low (Table 4), facilitating freer Cu^2+^ ion movement and thus increasing the mass transfer coefficient. However, Table 3 indicates that further increases in chalcone concentration reduce the reaction rate. This decline can be attributed to: (i) steric hindrance caused by chalcone molecules, (ii) increased solution viscosity, and (iii) adsorption of organic compounds on the zinc surface. The percentage acceleration of the cementation rate at 25 °C, calculated using the following equation:

12 $$\:\text{\%}\:\text{A}\text{c}\text{c}\text{e}\text{l}\text{e}\text{r}\text{a}\text{t}\text{i}\text{o}\text{n}\:=\left(\frac{k-\:K_{0}}{k_{0}\:}\right) \times 100$$

where *k* and *k₀* are the mass transfer coefficients with and without chalcone, respectively. The acceleration percentage follows the order: d > c > a > e > b.

**Table 6:** Percentage acceleration of the cementation rate at 25 °C.

Temp. °C	Conc. (molL^− 1^)	% Acceleration				
		a	b	c	d	e
25 °C	3.0 × 10^− 7^	23.94	12.11	24.93	33.38	21.97
	3.0 × 10^− 6^	17.46	4.51	18.31	19.58	17.18
	6.0 × 10^− 6^	12.54	− 4.37	13.66	15.49	12.11
	1.2 × 10^− 5^	6.62	− 8.17	7.75	10.42	3.80
	2.4 × 10^− 5^	− 1.69	− 11.41	− 2.96	− 0.42	− 2.96
	3.0 × 10^− 5^	− 10.42	− 20.00	− 9.86	− 6.62	− 11.55

The addition of chalcone derivatives led to a marked increase in the copper cementation rate, with compound D exhibiting the highest percentage acceleration (47.6%) compared to the blank. This enhancement is primarily attributed to the strong coordination ability of chalcone molecules with Cu^2+^ ions, which facilitates their transport and reduction on the zinc surface. This finding aligns well with recent studies that investigated organic modifiers for metal ion recovery. For instance, Li et al. reported a 40% increase in copper removal efficiency using bio-based ligands in a membrane adsorption hybrid system^8^. Similarly, Mukhtar et al. emphasized the role of electron-donating groups in chalcone structures in enhancing complications with metal ions, which supports the trends observed in our work^19^. However, unlike these studies that primarily utilized adsorption or membrane processes, the present study introduces a new application of chalcone derivatives in a cementation system, offering a simpler and more cost-effective alternative for Cu^2+^ recovery from aqueous solutions.

Furthermore, quantum chemical calculations using AM1 and PM3 methods provided molecular-level insights into the electronic distribution of heteroatoms in the chalcone molecules. Derivative D displayed higher negative charge densities on nitrogen atoms, suggesting stronger interactions with Cu^2+^ ions and thus explaining its superior performance. These findings are consistent with Liu et al., who noted a correlation between charge density distribution and removal efficiency in similar organic–metal systems^10^.At higher chalcone concentrations, a decline in reaction rate was observed due to increased solution viscosity, steric hindrance, and possible surface adsorption on zinc an effect that has also been reported in recent literature. Therefore, our study not only confirms known coordination behaviors of chalcones but also contributes a novel perspective by integrating them into the cementation process, supported by both experimental data and semi-empirical quantum analysis.

### Quantum calculations

**Fig. 5:**
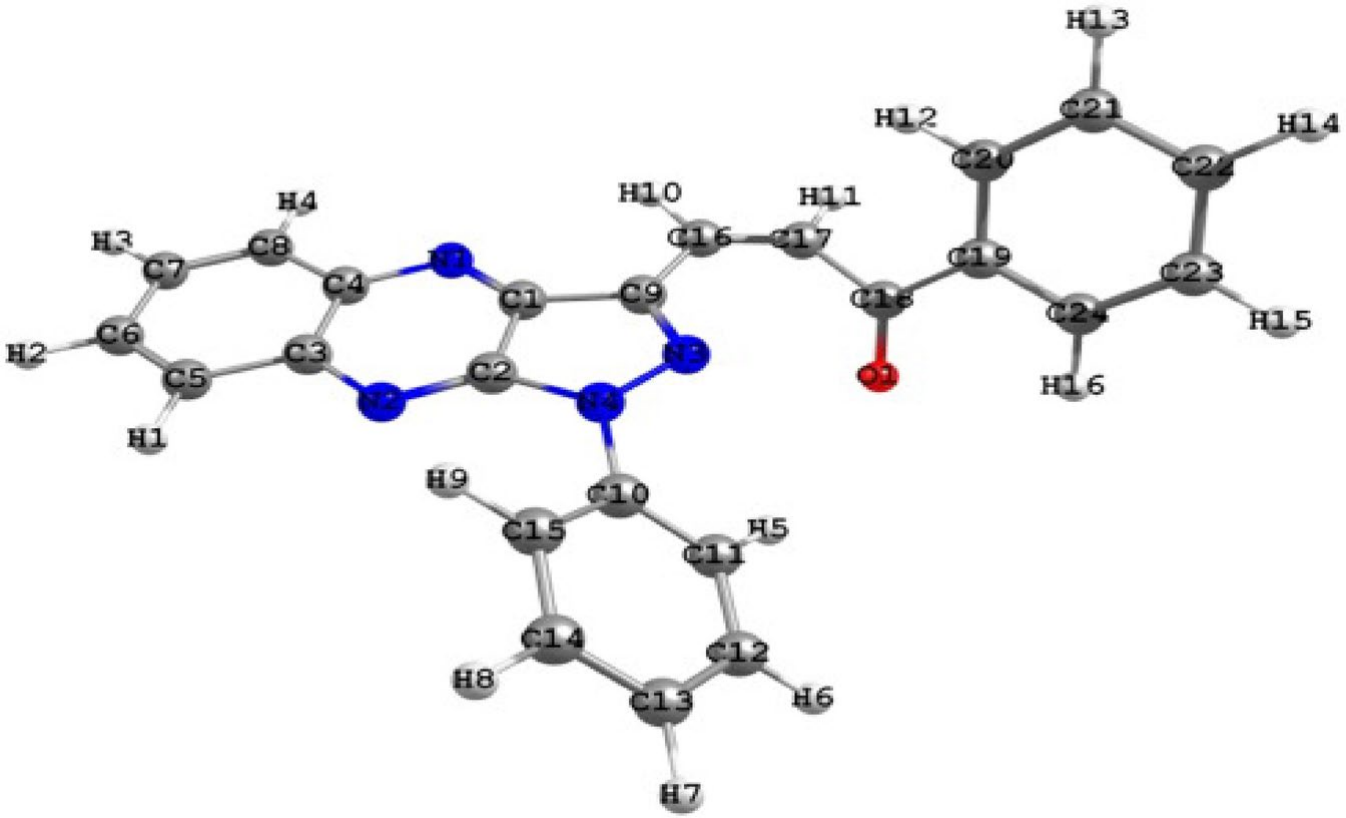
Show the coordination ability of Cu^2+^ ions with the negative centers (nitrogen and oxygen atoms) in the organic additives may explain the observed order of increasing percentage acceleration.

Figure 5 show the coordination ability of Cu^2+^ ions with the negative centers (nitrogen and oxygen atoms) in the organic additives may explain the observed order of increasing percentage acceleration. As the negative charge density on these active sites increases, the diffusion rate decreases, leading to a lower overall reaction rate. Table 7 presents the calculated nitrogen and oxygen charge densities of the investigated organic additives, obtained using two semi-empirical quantum chemical methods, Semi-empirical calculations (AM1 and PM3) were used to estimate the electron density on active atoms in chalcone molecules. Derivative D showed higher negative charge densities on nitrogen atoms, suggesting stronger coordination with copper ions. These electronic properties support the experimental findings and explain the superior performance of compound D^37^.

**Table 7:** Calculated charge densities of heteroatoms in the studied organic additives.

		N1	N2	N3	N4	O1
a	AM1	− 0.04635	− 0.104061	0.028935	− 0.109514	− 0.281517
	PM3	0.061438	− 0.027866	− 0.128718	0.269416	− 0.297417
b	AM1	− 0.046359	− 0.104310	0.028960	− 0.109662	− 0.283360
	PM3	0.061425	− 0.028148	− 0.128431	0.269018	− 0.298705
c	AM1	− 0.046324	− 0.103140	0.028704	− 0.109012	− 0.277795
	PM3	0.059174	− 0.024560	− 0.124427	0.266890	− 0.295962
d	AM1	− 0.046317	− 0.102967	0.028777	− 0.108926	− 0.275432
	PM3	0.059296	− 0.024212	− 0.125191	0.267755	− 0.293749
e	AM1	− 0.046372	− 0.104059	0.029078	− 0.109552	− 0.281402
	PM3	0.061493	− 0.027741	− 0.128977	0.269164	− 0.297194

The variation in percent acceleration for different chalcone derivatives can be attributed to the coordination ability of Cu^2+^ ions with the negative centers (particularly O and N atoms) of the organic additives. However, it is observed that the oxygen atom’s charge density plays a dominant role in determining the percent acceleration, while nitrogen atoms have a minor influence. This observation aligns with the Hard and Soft Acids and Bases (HSAB) theory (Koch), which posits that hard metal ions characterized by small ionic radii and high charge densities prefer to bind with hard base sites, such as oxygen^26^. Since Cu^2+^ is a hard acid, it tends to form more stable complexes with the oxygen atoms in chalcones. When electron-donating groups (e.g., –CH_3_) increase the electron density on the oxygen atom, the Cu–O bond strength increases, hindering the rate of Cu^2+^ ion diffusion and thus reducing the cementation rate. Conversely, electron-withdrawing groups such as Cl^−^ or Br^−^ decrease the oxygen’s electron density, weakening the Cu–O bond, promoting faster ion diffusion, and increasing the cementation rate. The order of increasing negative charge density on the oxygen atom, calculated using semi-empirical AM1 and PM3 methods, is as follows: d > c > a ≈ e > b. This order corresponds inversely with the reaction rate, supporting the proposed mechanism.

Furthermore, the dipole moment values of the organic additives (a-e) were calculated as 0.8818, 0.9000, 1.0439, 1.1000, and 0.8854 D, respectively. This indicates the following polarity order:

d > c > a > e > b, which directly correlates with the observed percent acceleration, suggesting that molecular polarity also plays a key role in facilitating the cementation reaction^21^.

### Morphology study

**Fig. 6:**
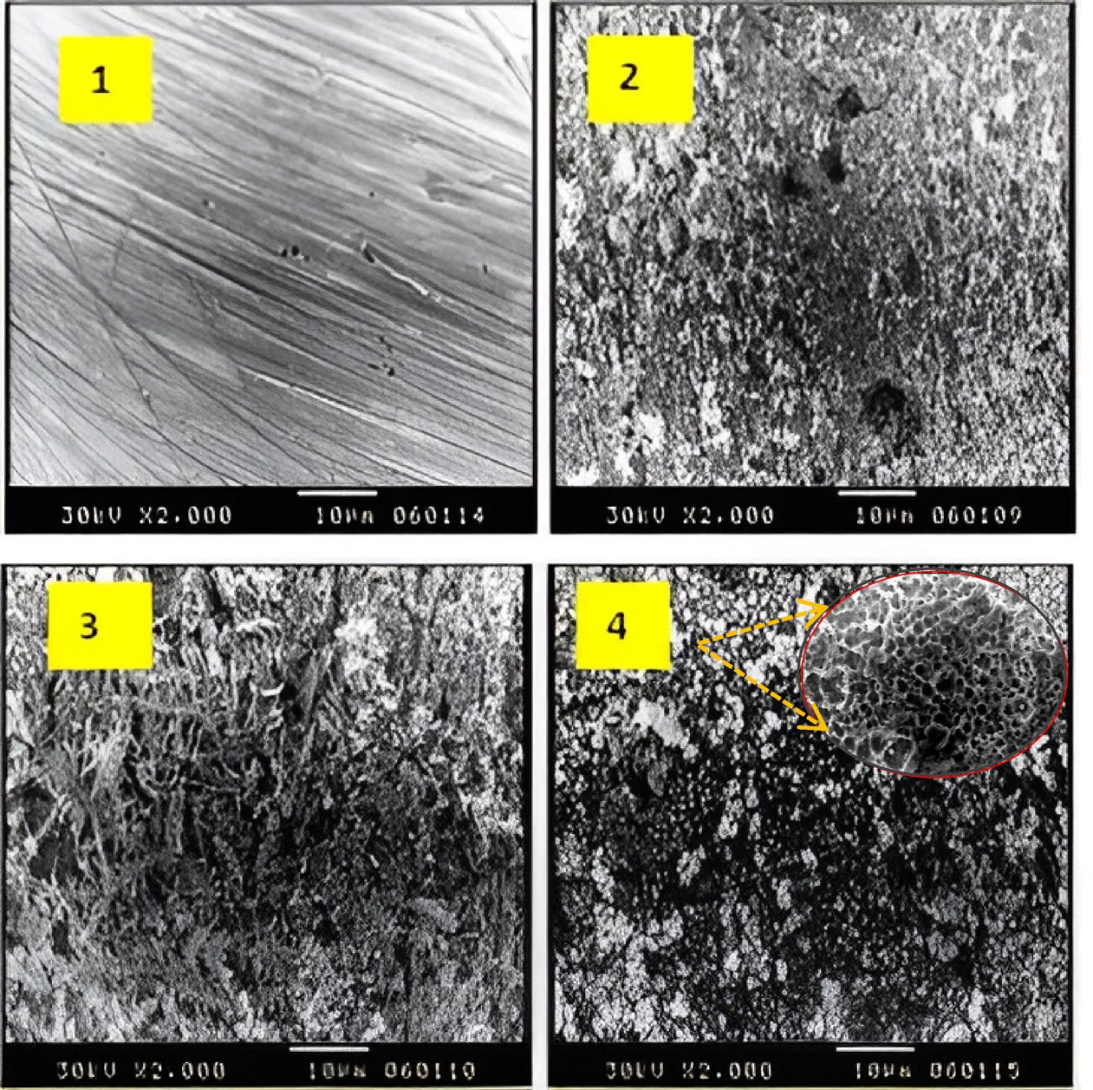
SEM morphology of zinc sheets (1–4) with an initial copper ion concentration of 100 ppm; chalcone concentrations of 1.2 × 10⁻⁵ mol L^−1^ for (a) and (d), respectively, showing samples before and after cementation.

Scanning Electron Microscopy (SEM) analysis was conducted to examine the surface morphology of zinc electrodes before and after the cementation process. Initially, the electrode surface exhibited visible scratches resulting from mechanical polishing with sandpaper. After 60 min of cementation in a 100 ppm Cu^2+^ solution, significant surface changes were observed, with irregular metallic copper deposits forming across the zinc surface. These deposits were rough and granular, with inconsistent grain sizes, indicating the formation of metallic copper through cementation. The observed surface morphology supports the previously proposed mechanism, wherein cementation occurs via a series of short-circuited electrochemical cells. In this mechanism, zinc functions as the anodic site and is oxidized, providing electrons for the reduction of Cu^2+^ ions. These copper ions are reduced and deposited on pre-existing copper sites, while the porous structure of the growing copper layer allows continuous diffusion of Zn^2+^ ions into the bulk solution, maintaining the electrochemical process. SEM images further revealed that the morphology of the cemented copper significantly changes in the presence of chalcone derivatives^38^. The deposits become more compact and dendritic, comprising porous grains with needle-like structures and a lower degree of discontinuity, leading to a rougher and more turbulent surface. This increase in surface roughness enhances the porosity and surface area, thereby facilitating the diffusion and electron transfer processes that drive cementation. Consequently, the presence of chalcone derivatives accelerates the reaction rate, as supported by the observed morphological changes. These findings confirm that copper cementation from copper sulfate solution can be effectively enhanced by suitable organic additives, producing copper powder with altered surface characteristics favorable for further applications. SEM analysis of the electrode (zinc plates) verified the surface alterations following the recovery procedure. The electrode surface had scratches from sandpaper polishing prior to cementation (Fig. 6). In contrast to the initial copper ion concentrations, metallic copper deposits were produced after 60 min of cementation (Fig. 6(2)). The metallic copper’s grain size is less consistent and somewhat roughened across the electrode surface at an initial concentration of roughly 100 ppm of copper ions. SEM images revealed the morphology of the deposited copper on zinc surfaces. Without additives, the deposits appeared uneven and sparse. In contrast, in the presence of chalcone D, the deposits were more uniform and dendritic, indicating improved coverage.

Further insights into the effect of chalcone derivatives on metal morphology were obtained by analyzing nickel deposits formed in the presence of different chalcone concentrations. As shown in Fig. 6(1–4), the morphology of nickel varied significantly between two chalcone concentrations: 1.2 × 10^− 5^ M (a) and 6 × 10^− 6^ M (d). The deposit in case (a) was found to be dense, well-adherent, and relatively smooth, whereas in case (d), the deposit exhibited a more porous and dendritic morphology with greater surface roughness. This rougher morphology in case (d) correlates with a higher cementation rate, in agreement with the previously discussed findings. The increase in surface roughness and porosity is attributed to hydrogen evolution during the reaction, where the attachment of hydrogen bubbles to the surface leads to localized pitting and microholes. These features enhance the electrochemical activity by increasing the available surface area for ion exchange.

**Fig. 7:**
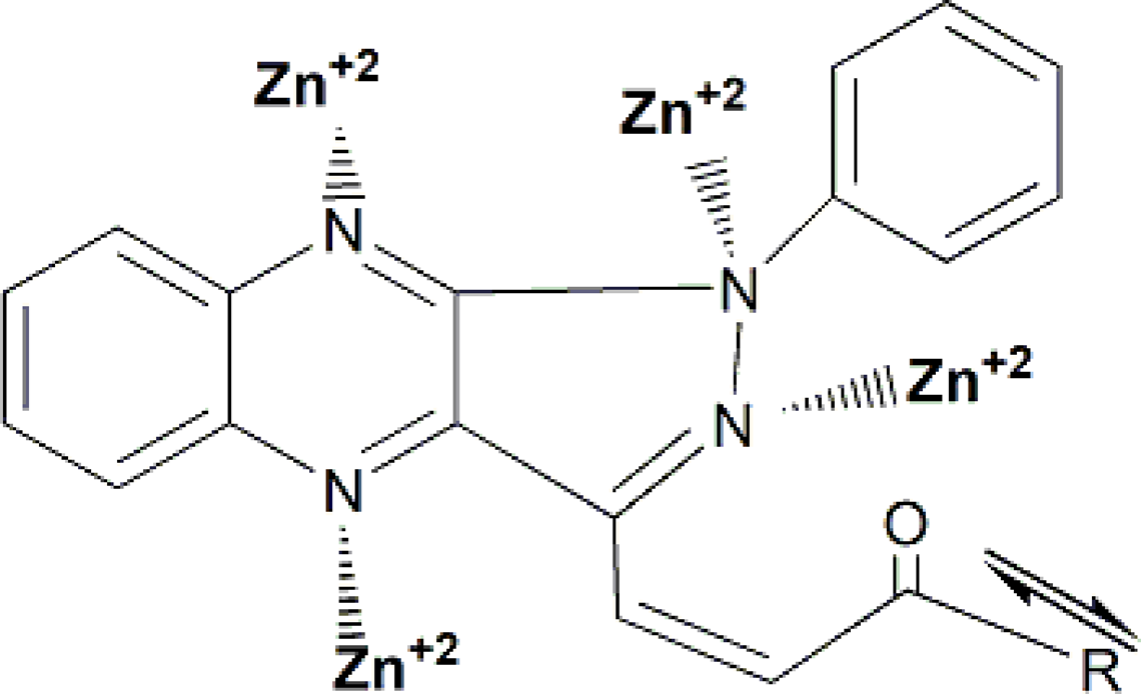
Skeletal representation illustrating the steric effect of the phenyl group.

Additionally, the electronic nature of the substituents on the chalcone structure plays a critical role in the cementation process. Electron-withdrawing groups such as chlorine (Cl) and bromine (Br) reduce the electron density on the oxygen atom, weakening the Cu–O bond and thereby facilitating ion diffusion and electron exchange in the reaction medium. This results in a higher cementation rate. Conversely, electron-donating groups like –CH₃ increase the electron density on nitrogen atoms, strengthening Zn–N interactions and thus slowing the reaction. The steric hindrance from bulky groups, such as the phenyl ring, further contributes to the reduced diffusion rate by obstructing access to the electrode surface. These observations are consistent with the morphological and kinetic data, and are illustrated in Fig. 7^37^.

### EDX spectroscopy

**Fig. 8:**
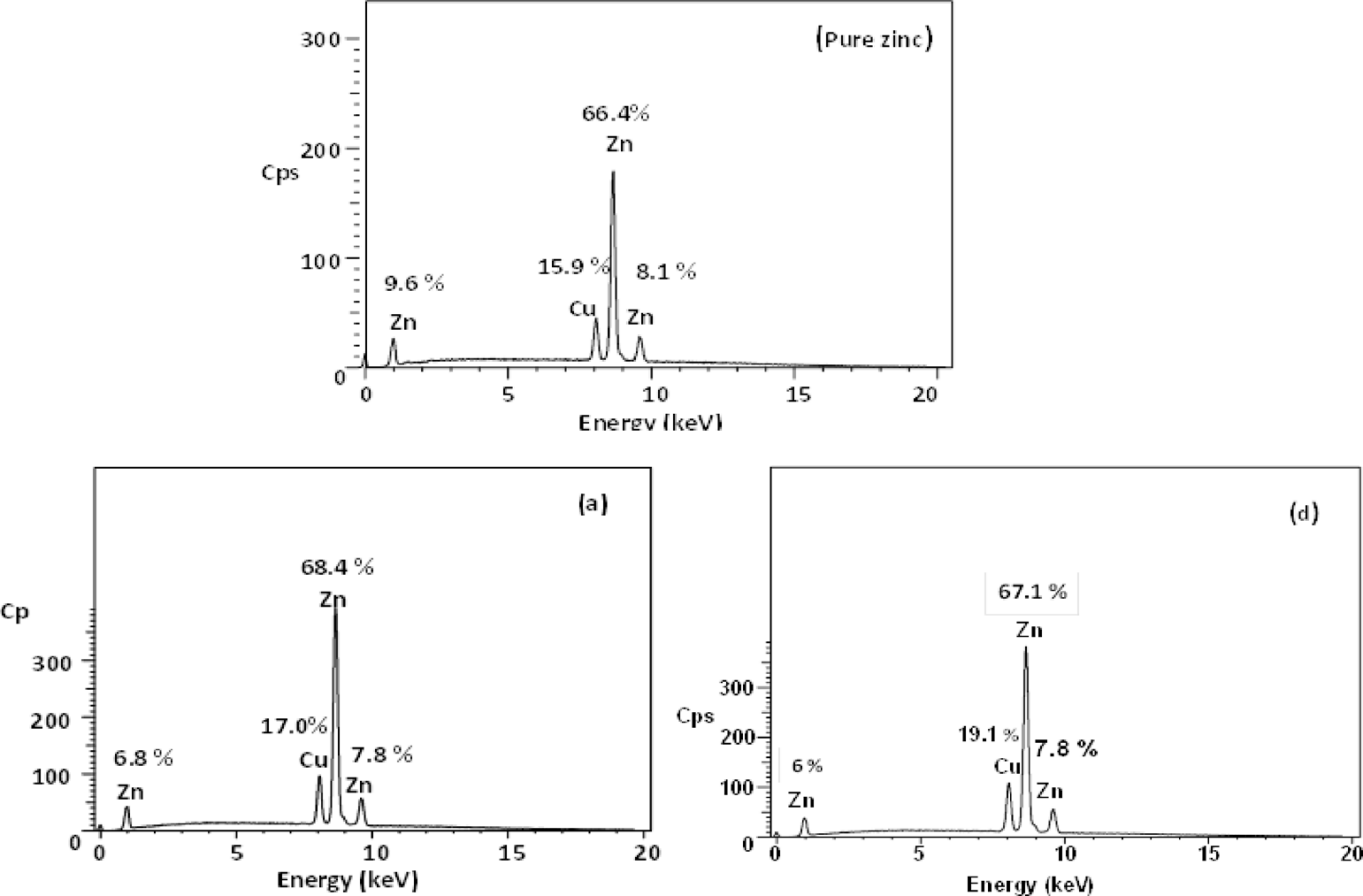
EDX profile analysis of zinc sheet immersed in Cu^2+^ solution, in the absence (a) and presence (d) of chalcones.

Energy-dispersive X-ray spectroscopy (EDX) was employed to analyze the elemental composition of the cemented metal deposits. As shown in Fig. 8 (samples of pure zinc, a, and d), the EDX analysis of the blank (chalcone-free) solution revealed a copper content of approximately 15.9% and a zinc content of 66.4%. This high zinc signal, alongside the copper, suggests that the copper deposit was not fully compact, allowing exposure of the underlying zinc surface. The porous nature of the copper precipitate was primarily inferred from SEM micrographs, which showed a dendritic and irregular morphology with visible voids consistent with porosity. Upon the introduction of chalcone derivatives, a noticeable increase in the copper content of the deposits was observed. Specifically, the copper content increased from 17.0% for compound (a) to 19.1% for compound (d). This trend confirms that the presence of chalcones enhances the cementation efficiency by promoting greater copper deposition. These findings align well with the kinetic results discussed earlier, further supporting the role of chalcones in accelerating the cementation process^39^.

### Cementation of Cu^2+^ from solution

To describe the mass transfer behavior during the cementation of Cu^2+^ ions from solution, empirical correlations involving dimensionless numbers were utilized. Specifically, the Sherwood number (Sh), Reynolds number (Re), and Schmidt number (Sc) were applied to establish a relationship that reflects the hydrodynamic and diffusion characteristics of the system. These dimensionless groups are defined as follows: the Sherwood number (Sh = kd/D), where k is the mass transfer coefficient, d is the characteristic length, and D is the diffusion coefficient; the Schmidt number (Sc = ν/D), where ν is the kinematic viscosity; and the Reynolds number (Re = Vd/ν), where V is the linear velocity.

A general empirical correlation of the form:

13 $$Sh\,=\,a{\text{ }}S{c^b}R{e^c}$$

Where a and c are empirical constants and b = 0.33. Other equation was used. Sherwood number (Sh), Reynolds number (Sc), Schmidt number (Re).

$$Sh\,=\,a{\text{ }}S{c^{0.{\text{33}}}}R{e^c}$$

$$Sh\,=\,0.{\text{89}}S{c^{0.{\text{33}}}}R{e^{0.{\text{49}}}} \quad {\text{for a}}$$

$$Sh\,=\,0.{\text{99}}S{c^{0.{\text{33}}}}R{e^{0.{\text{47}}}} \quad {\text{for b}}$$

$$Sh\,=\,0.{\text{81}}S{c^{0.{\text{33}}}}R{e^{0.{\text{46}}}} \quad {\text{for c}}$$

$$Sh\,=\,0.{\text{91}}S{c^{0.{\text{33}}}}R{e^{0.{\text{54}}}} \quad {\text{for d}}$$

$$Sh\,=\,0.{\text{83}}S{c^{0.{\text{33}}}}R{e^{0.{\text{47}}}} \quad {\text{for e}}$$

The overall correlation for all Chalcones which correlated by the equation:

$$Sh\,=\,0.{\text{81}}S{c^{0.{\text{33}}}}R{e^{0.{\text{47}}}}$$

## Conclusion

This study investigated the influence of pyrazolo-quinoxaline derivatives, functionalized with chalcone moieties, on the cementation rate of Cu^2+^ ions onto zinc substrates. The results demonstrated that the reaction rate increased with rising concentrations of the derivatives, lower solution viscosity, enhanced diffusion coefficients, and elevated temperatures. The cementation process followed first-order reaction kinetics, with diffusion identified as the rate-controlling step. The reaction acceleration was strongly influenced by both the electronic nature and the concentration of substituent groups attached to the heterocyclic core of the biomolecules. Additionally, the observed increase in rate with higher stirring speeds supported a diffusion-controlled mechanism, as confirmed by activation energy values. Among the tested chalcone derivatives (a-e), compound D exhibited the highest enhancement in copper cementation efficiency, achieving an acceleration percentage of 47.6%. This superior performance is attributed to its higher negative charge density on nitrogen donor atoms, as revealed by quantum chemical calculations, which likely facilitates stronger coordination with Cu^2+^ ions. SEM imaging further supported this finding, showing more extensive and uniform copper deposition on the zinc surface in the presence of compound D. These dendritic copper structures increased surface roughness and induced localized turbulence, further promoting mass transfer and accelerating the process. This finding highlights its potential for sustainable and efficient copper recovery strategies, particularly in environmentally conscious hydrometallurgical applications.

## Limitations and future scope

Our study demonstrates the potential of chalcone-enhanced zinc cementation for copper recovery, yet several limitations must be addressed. The chalcone concentration used may influence mass transfer dynamics, and the lack of Zn^2+^ quantification hinders precise stoichiometric validation. Additionally, the role of dissolved oxygen in zinc surface chemistry remains unexplored, and porosity assessments were limited to XPS or BET. Focusing solely on a single chalcone derivative restricts the generalizability of the findings.

Future research should prioritize evaluating the effects of dissolved oxygen to elucidate passivation behavior and kinetic influences. Measuring Zn^2+^ release via AAS will complete mass-balance assessments. Assessing the reuse of zinc substrates across multiple cycles will determine operational sustainability. Testing in real industrial effluents containing mixed metals (e.g., Ni^2+^, Fe^2+^) will verify selectivity and robustness. Implementing advanced surface analysis (XPS( will confirm deposit characteristics and porosity. Optimizing reaction parameters (temperature, stirring, viscosity, and dosing) and incorporating high-fidelity quantum calculations will provide deeper mechanistic insights and readiness for scale-up.

## Data Availability

The datasets generated and/or analyzed during the current study are available from the corresponding author upon reasonable request.
